# A simplified measure of burnout symptoms among paramedics - an exploratory analysis of a Hungarian sample

**DOI:** 10.1186/s40359-024-01518-x

**Published:** 2024-01-18

**Authors:** László Ivánkovits, Csaba Kazinczi, Krisztián Kocsis, Mona Stankovic, Zsuzsanna Koday, Zoltán Pető, Annamária Töreki

**Affiliations:** 1 Department of Emergency Medicine, University of Szeged, 6, Semmelweis Street, Szeged, 6725 Hungary; 2 Department of Neurology, University of Szeged, 6, Semmelweis Street, Szeged, 6725 Hungary; 3 Department of Clinical Psychology, Semmelweis University, 25, Üllői Street, Budapest, 1091 Hungary; 4 Department of Radiology, University of Szeged, 6, Semmelweis street, Szeged, 6725 Hungary; 5 Eötvös Lóránd Univesity, Egyetem tér 1-3, Budapest, 1053 Hungary

**Keywords:** Burnout, Paramedics, Explanatory analysis, Well-being

## Abstract

**Background:**

Burnout is still one of the leading mental health problems. According to research results over the past decades, healthcare workers, including paramedics, are considered a high-risk group. In concordance with these results, the available resources need to prioritize monitoring paramedics’ mental health.

**Methods:**

In our study, we investigated whether the available test batteries measuring burnout could be reduced while maintaining their effectiveness. We reduced the 21-item Burnout Measurement and the 8-item version of the Psychosomatic Symptom Scale using the data of 727 Hungarian paramedics. We selected the top four items of the questionnaires that were significantly correlated with the original Burnout Measure Index and the Psychosomatic Scale Index. The classification efficiency of the shortened list of items was based on the initial risk categories of the Burnout Measure and its sensitivity was analyzed using Binary Logistic regression and ROC curves. We then used Two-Step Cluster Analysis to test the ability of the shortened Burnout Measure Index to develop new risk categories. The reliability indicators were also explored.

**Results:**

The results show that the Burnout Measurement can be reduced to 4 items with a classification efficiency of 93.5% in determining the level of burnout. The 5-item reduction of the Psychosomatic Symptom Scale can classify subjects to the appropriate intervention level for burnout with an efficiency of 81.6%. The ROC analysis suggests that the shortened questionnaires have an excellent separative ability to discriminate between the initial risk groups. Three new risk categories were also identified as a result of the cluster analysis.

**Conclusion:**

The shortened scales may be proven effective in resource management, which could significantly quicken the assessment of burnout in the future. The abbreviated scale is also suitable for classifying subjects into risk categories. However, further research is needed to see whether the shortened scales can be used as a diagnostic tool.

**Supplementary Information:**

The online version contains supplementary material available at 10.1186/s40359-024-01518-x.

## Background

Over the past decades, numerous studies have been carried out on psychological well-being, especially burnout syndrome [1–3]. This complex phenomenon occurs when an individual is exposed to work stressors that simultaneously affect their mental, psychological, and somatic state as well as their overall performance [4, 5]. Researchers have also pointed out that burnout syndrome is more akin to psychological disorders associated with a decline in mood and performance, such as depression [6, 7]. It is important to highlight that the exact definition of burnout is still being debated today. The signs of burnout can be described by a combination of three main symptoms: emotional exhaustion, reduced personal accomplishment and depersonalization. In exhaustion, people feel emotionally drained, unable to cope, tired and enervated. Burnout causes people to disengage from their work, as they experience it as stressful and frustrating, while their performance also decreases. They find it hard to concentrate and their creativity levels drop. Another important consideration is how burnout is distinguished from depression. Although many symptoms overlap between the two conditions, such as fatigue, feelings of dejection and reduced performance, in most cases burnout symptoms are work-related, and the symptoms relieve when work stressors are removed whereas in depression, they are not exclusively work-related. However, burnout can develop into depression and lead to a range of symptoms, such as sleep disturbances, memory, attention and concentration problems, elevated cortisol levels [8–10]. The question logically arises, whether the symptoms develop simultaneously. In order to understand the temporality of burnout symptom, three widely accepted models exist. The phase model states that the first symptom is the depersonalization (cynical, indifferent attitude towards care recipients), because in services it might be necessary to detach at some level, but later it becomes depersonalization [11]. In contrast to the phase model, the process model states that the first symptom is the emotional exhaustion due high demanding work conditions. Emotional exhaustion followed by depersonalization and reduced personal accomplishment [12]. Based on the third model, emotional exhaustion develops first, but depersonalization and decreasing personal accomplishments occur simultaneously [13]. Both the symptoms of continuous, long-term stress and physical exhaustion have a negative impact on the motivation and productivity of the individual [14]. Although various professions are affected differently, it is generally accepted that healthcare workers are at higher risk in this respect [15]. Moreover, problems arising from burnout symptoms in healthcare professionals are common worldwide [16].

The medical staff is divided into several segments depending on the type of care. For instance, the paramedic staff treats emergency patients who are difficult to reach and require transport [17]; therefore, they are subject to considerable physical and mental demands. In addition, several studies confirm that symptoms of burnout in this area develop relatively rapidly; hence turnover is usually high [18, 19]. So far, the available measurement tools have focused less on burnout among paramedics, even though this population is affected by burnout in about 16–56% [20]. Questionnaires that measure burnout-related complaints aim to cover the full spectrum of symptoms differently [21]. Notably, some people may not be able to perceive the psychological symptoms of burnout directly or may find it difficult to articulate them, so an assessment of psychosomatic symptoms may be crucial [22–24]. According to the review of Reardon and colleagues (2020), burnout in paramedics can be summarized from 5 studies based on the Copenhagen Burnout Inventory and the Maslach’s Burnout Inventory [20]. The Maslach’s Burnout Inventory is a self-administered questionnaire, that measures the level of the main burnout symptoms, namely the emotional exhaustion, depersonalization and decreased personal accomplishment. The instrument consists of three sections with 7-7-8 items, and the participants have 7 answer options. Higher scores in the emotional exhaustion and depersonalization combined with lower scores in the personal accomplishment section indicate burnout [25]. The Copenhagen Burnout Inventory measures the level of burnout from personal, work-related or client-related perspective. The instrument contains 19 items, and participants can answer on a 5-point scale [26]. In order to assess the level of burnout, several other instruments can be used also. The Oldenburg Burnout Inventory assess the aspects of exhaustion and disengagement from work [27]. The Shirom-Melamed Burnout Measure aims to describe the level of physical fatigue and cognitive weariness using 6–6 items with a 7-point frequency scale [28]. These instruments assess the physical and emotional aspects of burnout in slightly different perspectives and consists of many items, which may still be challenging to complete due to unpredictable time management of paramedics. In addition to these questionnaires, it is important to mention another instrument, namely the Burnout Measure (BM) by Pines and Aronson [29], which is typically used in Hungary to measure healthcare professionals’ burnout [30–32]. The concept behind the BM was to create an inventory, that is available to use in many occupational fields. The BM determines the level of burnout in three main aspects, namely the emotional, physical and mental exhaustion. The emotional exhaustion determines the level of helplessness, hopelessness and entrapment. Items aiming to assess physical exhaustion consists of questions about the energy level, fatigue and weakness, whilst mental exhaustion assess the negative attitude towards work, one’s and life itself’. The BM determines the level of burnout using 21 items [33]. Both the BM and the questionnaires described above contain many items that are time-consuming and difficult to collect at the level of a large organization or institution. To overcome this, the 10-item, shortened version of the BM seems to be a suitable instrument [34]. Nonetheless, there may still be a need to obtain valuable results with further reduction in the number of items, as in the case of the WHO-5 questionnaire, which uses only five items [35]. An additional argument in favour of optimization is the high workload of the healthcare system, where resource management is also an essential factor, as burnout can lead to significant economic and efficiency losses, in addition to the psychological vulnerability of the individual [36, 37].

In present study, we investigate the degree of burnout among paramedics and how it differs from the general population. Additionally, we aim to reduce the length of the questionnaires used in Hungary for measurement of burnout and psychosomatic symptoms, while maintaining their high efficiency. We hypothesize that there are items in the surveys we use that allow for shortening, optimizing the measurement of burnout.

## Methods

Present study is a final phase of a longitudinal study on mental health of paramedics in Hungary conducted between 2016 and 2021. The following measurement started in 2020 using a quantitative method, both paper-based and online via Google Survey. Both test batteries contained the same demographic questions, questionnaires and ethical approval. The data collection lasted approximately two months. The researchers did not exercise control over the response process. However, by completing and submitting the questionnaire, the subjects consented to the study, which adhered to the guidelines of the Helsinki Committee Code of Ethics. The study was conducted with the permission of the Regional Medical and Research Ethics Committee of the University of Szeged (number: 29,640). The sample consisted of people aged 18 or over working full-time for the National Ambulance Service of Hungary (OMSZ).

### Instruments

#### The Burnout measure (BM)

The level of burnout was assessed using the questionnaire developed by Pines & Aronson (1981). The questionnaire focuses on items identified in previous research on burnout syndrome. Each item was scored on a seven-point Likert scale (1 = never, 2 = once or twice, 3 = rarely, 4 = sometimes, 5 = often, 6 = usually 7 = always), rating symptoms that have occurred in the past 12 months. Answers were then categorized the following way: between 1 and 2 points state of constant euphoria; between 2 and 3 points no intervention needed; between 3 and 4 points need for change; above 4 points requires intervention [38].

#### Psychosomatic symptom scale (PSS)

The somatic background of burnout was assessed using a Hungarian-validated version of the Psychosomatic Symptom Scale [39]. Each symptom is scored on a scale from 0 to 3 (0 = never, 1 = rarely, 2 = occasionally, and 3 = often). According to the original validation in a standard sample, from a total of 21 points, women scored an average of 6.1 points and men 5.0 points.

### Additional questions

Before taking the primary questionnaires, general questions were asked about: the atmosphere at work; working conditions; whether they take on side jobs; and subjectively assessed health status. We also asked respondents to evaluate their work-life balance; whether they had enough time off; how often they have been involved in a traumatic accident at work; what were the most distressing decisions they had to make in their work as a paramedic; how they dealt with difficulties at work; whether they discussed their problems at home; and how they dealt with stresses that also effected their families.

### Sociodemographic characteristics

The following factors were asked of the study participants: gender, age, highest level of education (primary education, high school education or university), place of employment (county) and status within the paramedic workforce, residence and marital status.

### Statistical analysis

Data were analyzed using the Statistical Package for Social Sciences (SPSS 25.0 for Windows, IBM Corporation, USA), where the significance level was set at .05. We used descriptive statistics to determine the characteristics of the sample. The normality was tested in all cases using the Shapiro-Wilk test. Due to non-normal distributions, non-parametric tests were performed. Reliability was determined by calculating Cronbach’s alpha. Mann-Whitney, Kruskal-Wallis and Chi-squared tests were used for between-group comparisons. During the analysis, we first filtered for the role in the paramedic workforce, excluding respondents who gave the status ’OTHER’. We then examined the reliability of the questionnaires taken. In this case, we only went further in the analysis if Cronbach’s alpha indicators were adequate. We then calculated the Burnout Index (BI) of BM to determine the degree of burnout by summing up the 21 items, after recoding the positively phrased items [33, 40]. Finally, we categorized the participants into predefined categories in the questionnaire [30–32]. While the original instrument discriminates four categories, we only distinguished between low-risk (0–3 points) and high-risk (above 3 points) groups. We then determined which of the items of BM and PSS correlated best with the BI. The methodological consideration was to find the most relevant and well-differentiating items. The two questionnaires contain 29 items (BM: 21; PSS: 8) that we correlated with the BI using Spearman correlation [41, 42]. The results were ranked according to Spearman’s rho, and the 5–5 items considered the most significant are used as one of the bases for the subsequent analysis. The classification reliability of the selected items was later tested by binary logistic regression (using “enter” and “forward conditional” methods) between the two risk groups. In choosing the most optimal model, we considered the number of items and the percentage of correct classification. On this basis, we determined the most effective model, defined as the ratio of the classification percentage and the number of items. Here, the model with the highest index was chosen for further analysis. Based on the elements of the selected model, a shortened burnout index (SBMI) was created, and its correlation with the BI was examined. Subsequently, we also determined Cronbach’s alpha and sensitivity (ROC analysis) of the SBMI. Then we used Two-Step cluster analysis (automatic, BIC model) to examine the characteristics of the categories created by the SBMI. The cut-off scores for the categories were determined using the Youden Index, calculated using the formula: (Sensitivity + Specificity)-1 [43]. If no psychosomatic complaints appear within the most effective model, it is abbreviated separately and appended to the items that primarily examine burnout.

### Sample characteristics

The questionnaire was completed by a total of 815 respondents, with 727 (*N* = 727) remaining in the sample after pre-screening. A total of 637 men and 90 women remained in the sample. Age mean of the total sample was 40.02 years, 40.77 years for men and 34.69 years for women. Most of the respondents (62,17%) had completed secondary school education. The BI has a median of 2.33 points, with 2.33 points for men and 2.35 points for women. The sample was homogeneous in this respect, with no significant difference according to the Mann-Whitney test performed (Z=-1.292, U = 26255.0, *p* = 0.196). For PSS, the average score was 9.04, 8.88 for men and 1.14 for women. These scores were significantly higher than the mean scores of the originally validated test. In addition, the correlation between the burnout index and the psychosomatic index was found to be strong (rho = 0.735; *p* < 0.0001) (*For sample characteristics*, see Table 1.).

**Table 1:** Descriptive statistics of the sample. Results show that the main characteristics do not meet the criteria of normal distribution

	N	Minimum	Maximum	Mean (Median)	SD	Test of normality (p)
Age	727	19.00	64.00	40.02 (40.00)	10.22	< 0.0001
Years of Work	727	0.15	45.00	14.77 (12.00)	11.10	< 0.0001
BM Index	727	1.00	7.00	2.70 (2.33)	1.20	< 0.0001
PSS Index	727	0.00	24.00	9.04 (9.00)	4.90	< 0.0001
		**Sex**		**Education**		
		Male	Female	Primary education	Secondary education	Higher education
	N	637	90	28	452	247
	Percentage (%)	85.88	14.12	3.85	62.17	33.98
		**Marital State**				
		Single	In a relationship	Married	Divorced	Widow
	N	88	234	438	53	2
	Percentage (%)	10,8	28,7	53,7	6,5	0,2
		**Job Title**				
			Emergency physician	Paramedic officer	Emergency nurse	Ambulance driver
	N		40	173	343	171
	Percentage (%)		5,5	23,8	47,2	23,5

### Preliminary analysis and correlations

Before correlation analysis, we first examined the normality and reliability of each item in the questionnaire. The BM had a Cronbach’s alpha of .813, and the PSS had a Cronbach’s alpha of .898. Without exception, the items deviated from the normal distribution. Subsequent tests revealed that the following five items were found to be the most significantly correlated with the BI: being emotionally exhausted (rho =.833, p < 0.001); being ’wiped out’ (rho = 0.831, *p* < 0.001); feeling rundown (rho = 0.822, *p* < 0.001); feeling hopeless (rho = 0.777, *p* < 0.001); feeling ’burned out’ (rho = 0.766, *p* < 0.001). The Cronbach’s alpha coefficients for the selected five items were re-examined and were found to be 0.920, meaning high reliability. The items of the PSS also showed a correlation with BI: feeling weak and tired (rho = 0.649, *p* < 0.001); sleeping problems (rho = 0.595, *p* < 0.001); stress diarrhoea (rho = 0.490, *p* < 0.001); palpitation (rho = 0.461, *p* < 0.001); backache (rho = 0.439, *p* < 0.001). For somatic problems, Cronbach’s alpha was 0.748, assuming good reliability. The psychological and somatic items were combined, giving a total of 10 items, and then the reliability indicators were re-tested here, with a value of 0.901.

### Binary logistic regression

Binary logistic regression was used to test the predictive accuracy of the selected items between the low and high-risk groups. First, the five BM items were examined from the point of view of classification. Using the Enter method, efficacy was found to be 93.8% with a significant model (χ2 (df 5, Ntotal: 727, low-risk group: 466, high-risk group: 216) = 708.576 *p* <.0001). Applying the forward conditional method, the efficiency was 93.5%, and one item (*Feeling ’burned out’*) was not required in the model (χ2 (df 4, Ntotal: 727, low-risk group: 465, high-risk group: 215) = 705,173 *p* < 0.0001). For PPS, using the enter method, an accuracy of 81.6% is obtained (χ2 (df 5, Ntotal: 727, low-risk group: 436, high-risk group: 157) = 334,401 p<. 001), also for the forward conditional method (χ2 (df 5, Ntotal: 727, low-risk group: 436, high-risk group: 157) = 334,401 *p* < 0.0001). In the case of 10 items, using the enter method, we obtained an accuracy of 94.5% (χ2 (df 10, Ntotal: 727, low-risk group: 469, high-risk group: 218) = 731,359 p<. 001), while the forward conditional method yielded 93.9% (χ2 (df 10, Ntotal: 727, low-risk group: 464, high-risk group: 219) = 726,346 *p* < 0.0001). Therefore, the four-batch BM model seems to be the optimal choice based on the given ratio. Combining the four items created a new burnout index for further analysis (SBMI). For the shortened version of the questionnaire see Supplementary material 1.

### ROC analysis and clustering

**Fig. 1:**
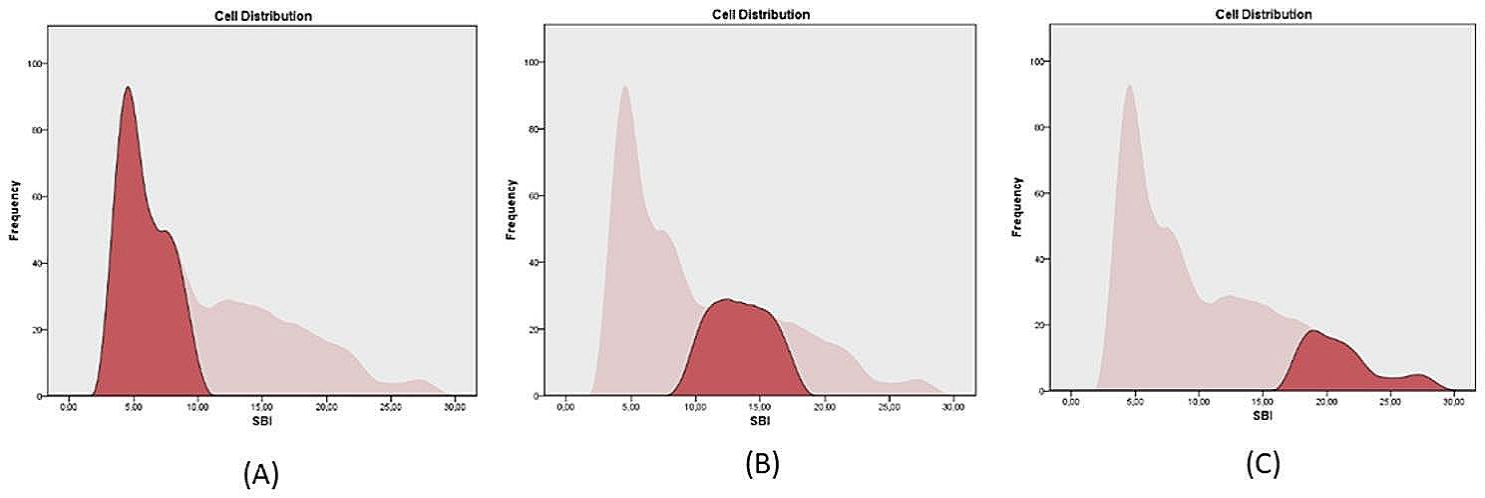
Results of the cluster analysis. Using SBMI, three risk categories were identified (**A**) low risk; (**B**) medium risk; (**C**) high risk. The resulting clusters have good separation indicators

**Fig. 2:**
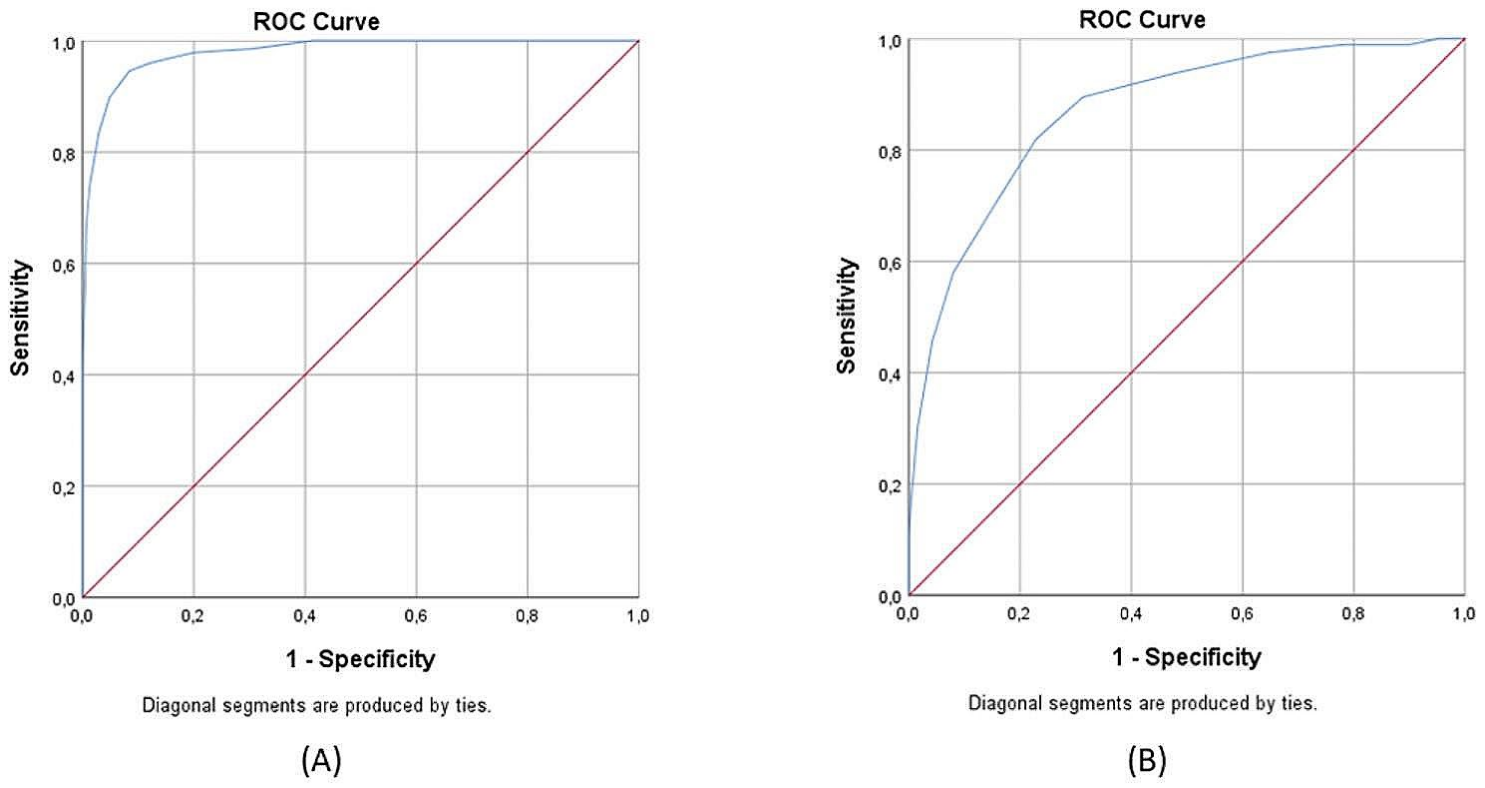
Results of the ROC curves. Comparing SBMI (**A**) and SPSSI (**B**), the results show that SBMI has an excellent level of sensitivity and specificity (AUC_SBMI_=0.979), while SPSSI (AUC_SPSSI_=0.871) also obtained good markers

The Cronbach’s alpha for the four selected items was excellent (0.905) and correlated well with the BM (rho = 0.936) and PSS (rho = 0.683). The ROC curve was calculated to confirm the sensitivity and specificity tradeoff further. The analysis also shows that SBMI has an excellent separation ability concerning the assessment of the original risk groups (AUC_SBMI_ =. 979; 95% CI: 0.971 − 0.988) (For ROC curves, see Fig. 1). We also examined whether the SBMI can distinguish between different risk groups. Using the Two-Step clustering method (BIC, automatically determined clusters), we obtained three well-distinguishable groups [High risk (M = 21.18, SD = 2.91); Moderate risk (M = 13.42, SD = 2.19); Low risk (M = 5.81, SD = 1.72)] *(For cluster characteristics see* Fig. 2; Table 2). For the low and moderate risk and the moderate and high-risk categories, the most optimal cut-off values would be 9.5 and 17.5 points respectively, but the test results are calculated with whole points. Thus, the Youden-index of the two closest integer scores [27,28,41] was determined, and the score with the better indicator was chosen as the cut-off value (J_10_ = 0.704 vs. J_9_ = 0.679; J_18_ = 0.964 J_17_ = 0.798). Since the items of PSS did not appear in the current model, the selected five items were used to differentiate burnout levels. In this case, we also examined Cronbach’s alpha values of the shortened PSS index (SPSSI) (0.748) and the correlation between BI (rho = 0.744) and SBMI (rho = 0.698). Furthermore, the ROC analysis results show that SPSSI has good sensitivity and specificity (AUC_SPSSI_ =. 871; 95% CI: 0.845 − 0.897).

**Table 2:** Descriptive statistics of the clusters based on SBMI

	Low Risk		Moderate Risk		High Risk		
	(n)	Mean (SD)	(n)	Mean (SD)	(n)	Mean (SD)	Group differences *(p)*
Age	400	40.02 (10.32)	206	39.84 (10.34)	114	40.30 (9.66)	0.919
Years of Work	400	14.12 (11.42)	206	15.30 (10.95)	114	16.10 (10.08)	0.021
BM Index	400	1.85 (0.42)	206	3.24 (0.54)	114	4.84 (0.73)	< 0.0001
PSS Index	400	6.46 (3.64)	206	11.00 (3.51)	114	14.75 (4.52)	< 0.0001
SBM Index	400	5.81 (1.72)	206	13.42 (2.19)	114	21.18 (2.91)	< 0.0001
SPSS Index	400	4.42 (2.52)	206	7.71 (2.45)	114	10.24 (2.90)	< 0.0001
Sex (n) (Male/Female)	358/49		182/24		97/17		0.675
Education (n) (Primary/Secondary/Higher)	15/213/139		10/128/68		3/71/40		0.890

## Discussion

Our research aimed to create a shortened scale from the available questionnaires adapted to the characteristics of healthcare workers, specifically paramedics. We wanted to establish a quick yet reliable and predictive set of questions, that can be used to differentiate between different burnout levels, which may be a helpful tool to resource management. Our sample proved suitable for this purpose, as our BM showed a significant increase in our sample compared to the standard level of burnout, with 33% showing signs of burnout based on the original questionnaire and 44% on our shortened version. In the context of burnout, we also aimed to find any predictor importance of psychosomatic complaints combined with the items of BM, which would allow better prediction. Based on the results, we concluded that the level of burnout assessed by our test battery, including 29 items, could be shortened to four items, allowing a classification accuracy of 93.5% compared to the original questionnaire. However, psychosomatic symptoms were not included in these four items. The results thus also indicate that, although psychosomatic complaints appear in burnout, the main problem area is primarily psychological and is significantly related to the following factors: being emotionally exhausted; being ‘wiped out’; feeling rundown; feeling hopeless; feeling ‘burned out’ [44, 45]. The strength of the correlates also suggests that subjects may perceive other emotional and mental states first but may not yet be aware that these are directly related to burnout. These findings support the research of Kremer-Hayon (1985) and Seidler (2014), which points out that emotional exhaustion and rigidity underlie burnout and fundamentally affect the resilience of individuals [46, 47]. However, it is also essential to remember that some people find it more challenging to articulate their psychological difficulties, and (psycho)somatic complaints are much more likely to be the leading signs of burnout [48]. Present study has identified the five most common psychosomatic symptoms associated with burnout. As a result, a shortened scale was also created here, which classifies people into some level of burnout with an accuracy of 81.6% and has good sensitivity.

Consequently, one of the results of our research is that the size of the questionnaire on burnout can be reduced, and this does not imply a significant reduction in classification. Still, it should also be possible to include psychosomatic symptoms. In the categories created by the SBMI, age, gender, education and type of job did not seem to show significant differences, i.e. burnout can occur at any age or gender, regardless of employment and education. However, those working as paramedics for longer are at higher risk. In parallel, psychosomatic complaints are significantly higher in the high-risk burnout groups, and their general health status is perceived to be lower.

The shortened scale has classification properties and could be used as a measurement tool; additionally, it draws attention to the fact that there are probably distinct stages in the process that allow for more targeted intervention. In case of paramedics, a shortened version of the test battery seems particularly useful, as it is possible to assess their level of burnout after answering four questions. This would allow for the actual treatment of burnout and its prevention. For example, the first step in preventing and managing burnout would be identifying which factors are responsible for developing symptoms in a given group [49, 50]. Thus, research has an essential role in helping develop appropriate methods, solutions and strategies, thereby improving the well-being of healthcare workers and the high level of care provided. However, the perception of burnout as a decrease in engagement cannot be determined from these factors; presumably, it may still be more of a mental state close to depression, which does not directly reflect the individual’s attitude. We consider that it may be worthwhile to assess the level of stress on the employee precisely, the level of professional and social recognition, and to further explore links with the level of compensation. A further factor is the emergence of difficulties affecting society in general, such as SARS-CoV-19, which was in an emergent stage in Hungary at the time of data collection, suggesting that burnout is exacerbated in a health crisis following long-term stress. Furthermore, paramedics leaving the service further reduces the number of staff members on active duty, so the workload is much more concentrated in their case [51].

### Limitations

Concerning the limitations of the research, it is important to highlight at least two factors. One is that the results are primarily based on a Hungarian sample, and their interpretation of other countries’ healthcare systems requires caution. Furthermore, the study was conducted in the early stages of the SARS-CoV-19 epidemic, which may have already shown signs of a newly emerging burden, and the additional hardships were not compared. In addition, our study did not include psychopathological factors (e.g. depression) that would provide additional information regarding the measurability of this phenomenon. There is also the question of the ecological validity of the shortened questionnaire in terms of whether it measures burnout as effectively as the original 21-item version. Further research is needed to investigate this question with a new sample, comparing the effectiveness of both the shortened and the original questionnaire.

## Conclusion

In present research, we sought to determine whether we could measure the perceived level of burnout among paramedics using a shorter and quicker questionnaire. When analysing the data, we found that the sample had higher than normal levels of burnout. The reduction of the original 29-item test battery to 4 items did not significantly reduce its classification efficiency, with 93.5% accuracy in classifying respondents into one of the categories defined by the original questionnaire. It was also pointed out that psychosomatic indicators are crucial for burnout, so we selected the five most correlating items. Accordingly, we constructed a short questionnaire with a classification efficiency of 81.6% to perform an effective classification even based on physical symptoms. The reliability indicators of the generated inquiries are excellent, and they also show a high correlation with the original questionnaires. These features allow for an efficient and quick self-assessment, convenient for paramedics.

### Electronic supplementary material

Below is the link to the electronic supplementary material.

**Supplementary Material 1:** The Shortened Burnout Scale for paramedics

## Data Availability

Data available on request from the authors.

## Abbreviations

BM: Burnout Measure
BI: Burnout Index
PSS: Psychosomatic Symptom Scale
SBM: Shortened Burnout Index
SPSSI: Shortened Psychosomatic Symptom Scale Index
