# Supplementary material for: A simplified measure of burnout symptoms among paramedics - an exploratory analysis of a Hungarian sample
Source: BMC Psychol. 2024 Jan 18;12:37. doi: 10.1186/s40359-024-01518-x (PMC10797803; doi:10.1186/s40359-024-01518-x)
Supplement: Supplementary file 1 — Supplementary Material 1: The Shortened Burnout Scale for paramedics [file 40359_2024_1518_MOESM1_ESM.docx]

**Shortened Burnout Scale for Paramedics (ENG)**

| **How often have you experienced the following symptoms in the last 12 months? Use the scale below!**  1=never, 2=once or twice, 3=rarely, 4=sometimes, 5=often, 6=usually, 7=always. | | | | | | | |
| --- | --- | --- | --- | --- | --- | --- | --- |
|  | **1** | **2** | **3** | **4** | **5** | **6** | **7** |
| Being emotionally exhausted |  |  |  |  |  |  |  |
| Being ’wiped out’ |  |  |  |  |  |  |  |
| Feeling rundown |  |  |  |  |  |  |  |
| Feeling hopeless |  |  |  |  |  |  |  |

**Categories:**

**Over 18 points:** High risk

**Between 10-17 points:** Moderate risk

**Between 0-9 points:** Low risk
